# The Anti-Inflammatory Properties of *Citrus wilsonii* Tanaka Extract in LPS-Induced RAW 264.7 and Primary Mouse Bone Marrow-Derived Dendritic Cells

**DOI:** 10.3390/molecules22071213

**Published:** 2017-07-19

**Authors:** Liping Cheng, Yujie Ren, Dingbo Lin, Shu’ang Peng, Bo Zhong, Zhaocheng Ma

**Affiliations:** 1 College of Horticulture and Forestry Sciences, Key Laboratory of Horticultural Plant Biology, Huazhong Agricultural University, Ministry of Education, Wuhan 430070, China; chenglp@webmail.hzau.edu.cn (L.C.); ganjuli_2002@mail.hzau.edu.cn (S.P.); 2 College of Life Sciences, Medical Research Institute, Wuhan University, Wuhan 430072, China; yujieren1002@sina.cn (Y.R.); zhongbo@whu.edu.cn (B.Z.); 3 Department of Nutritional Sciences, Oklahoma State University, 419 Human Sciences, Stillwater, OK 74078, USA; dingbo.lin@okstate.edu

**Keywords:** citrus, anti-inflammation, RAW 264.7 macrophages, primary bone marrow-derived dendritic cells

## Abstract

‘Zhique’ (*Citrus wilsonii* Tanaka) is a traditional Chinese medicine. Its fruits have been used to treat inflammation-related symptoms, such as cough and sputum, though the underlying mechanism remains poorly understood. The aim of this study was to investigate the anti-inflammatory properties of ‘Zhique’ pulp extract (ZQE) in lipopolysaccharide (LPS)-induced RAW 264.7 macrophages and primary mouse bone marrow-derived dendritic cells (BMDCs). The flavonoid profiles of the ZQE were determined by high performance liquid chromatography. The anti-inflammatory activity was evaluated in LPS-induced inflammatory RAW 264.7 macrophages and BMDCs through enzyme-linked immunosorbent assay, quantitative real-time polymerase chain reaction, and Western blot assays. Naringin was a predominant flavonoid occurring in ZQE, followed by eriocitrin, hesperidin, neohesperidin, rhoifolin, naringenin, and poncirin. ZQE exhibited a very low cytotoxicity in LPS-stimulated RAW 264.7 macrophages. Meanwhile, ZQE significantly inhibited the production of prostaglandins E2 and secretion of cyclooxygenase-2 protein in LPS-stimulated RAW 264.7 macrophages, and markedly suppressed the mRNA expression of inflammatory mediators, such as cyclooxygenase-2, tumor necrosis factor alpha, interleukin-1 beta (IL-1β), and IL-6 in LPS-induced RAW 264.7 macrophages and/or primary BMDCs. The ZQE inhibited the inflammatory responses in RAW 264.7 macrophages and BMDCs triggered by LPS. The results suggested that ‘Zhique’ has a high potential as a novel therapeutic agent to treat chronic inflammatory diseases.

## 1. Introduction

*Citrus wilsonii* Tanaka (Xiangyuan in Chinese), belongs to the citrus genus in plant taxonomy [1]. It is called ‘Zhique’ in the region of the Southern Qinling Mountains in China [2]. It is recorded that the dried fruit of *Citrus wilsonii* Tanaka fruit is one of the Fructus Citri, which is a traditional Chinese medicine in the Chinese Pharmacopoeia [3]. Fructus Citri exerts the pharmacological role of evacuating the liver “qi” stagnation, regulating “qi” by alleviation of mental depression, and reduction of phlegm. The dried ‘Zhique’ fruits have been used to treat stagnated qi in the liver and stomach, chest and rib pain, epigastric abdominal distention and fullness, vomiting and belching, and coughing and phlegm for many years. Meanwhile, *Citrus wilsonii* Tanaka is also considered as Fructus Aurantii (zhiqiao) in Citrus herbal medicine [4]. Fructus Aurantii has a similar function compared to Fructus Citri in curing chest tightness, “qi” stagnation, abdominal fullness pain, indigestion, phlegmy throat, and organ sagging [3]. Mechanistically, inflammation is a common cause of some of these diseases mentioned above, such as coughing and phlegmy throat [5,6].

Citrus fruits are rich in flavonoid constituents. It has been reported that flavonoids are the main chemical compounds in the literatures involved in Citrus herbal medicine; for instance, Fructus Aurantii has been identified to be rich in the flavonoid *O*-glycoside [7]. *Citrus wilsonii* Tanaka contains large amounts of flavonoids, such as naringin, eriocitrin, neoeriocitrin, rhoifolin, and melitidin [8,9]. An accumulating line of evidence shows that flavonoids exhibit the roles in antioxidant, anti-inflammatory, anti-diabetic, anti-adipogenic activities [10,11]. Meanwhile, growing evidence also indicates that the flavonoids in citrus play a vital role in the treatment of chronic inflammatory diseases, such as obesity, atherosclerosis, Type 2 diabetes mellitus, cardiovascular disease, rheumatoid arthritis, and even cancer [12,13,14]. Obviously, those studies on phytochemical analysis could provide valuable information for the quality control of Fructus Citri and/or other traditional Chinese medicines. Nevertheless, the efficacy and the underlying mechanism of *Citrus wilsonii* Tanaka or Fructus Citri or their extracts it remains poorly understood in relation to health and diseases, due to a lack of mechanistic studies in inflammation [9].

Inflammation is a natural physiological and immune response to damage or infection in host tissues and/or cells. Mononuclear phagocytes, as the first line of defense, detect damage- or pathogen-associated molecular patterns, trigger a series of signaling cascades, and induce the expression of various pro-inflammatory mediators and cytokines, including tumor necrosis factor-alpha (TNF-α), interleukin-1beta (IL-1β), and interleukin-6 (IL-6) [15,16]. Lipopolysaccharide (LPS), a bacterial endotoxin, can induce inflammatory reactions through stimulation of secretion, and through increasing the expression of inflammatory mediators and cytokines in these immune cells [17]. LPS-induced RAW 264.7 macrophages and BMDCs have been widely used as in vitro models in studies related to inflammation or immunization [18,19,20].

Therefore, the present study aimed to investigate the chemical features of the extract of ‘Zhique’ pulp (ZQE) and assess its anti-inflammatory properties in LPS-induced RAW264.7 macrophages and primary BMDCs. The flavonoid profile of ZQE was identified with qualitative and quantitative analyses, and the inhibitory effects of ZQE on inflammatory mediators, and cytokines, such as COX-2, TNF-α, IL-1β, and IL-6 were analyzed in LPS-induced RAW 264.7 macrophages and primary BMDCs.

## 2. Results

### 2.1. Profile of the ZQE

**Figure 1:**
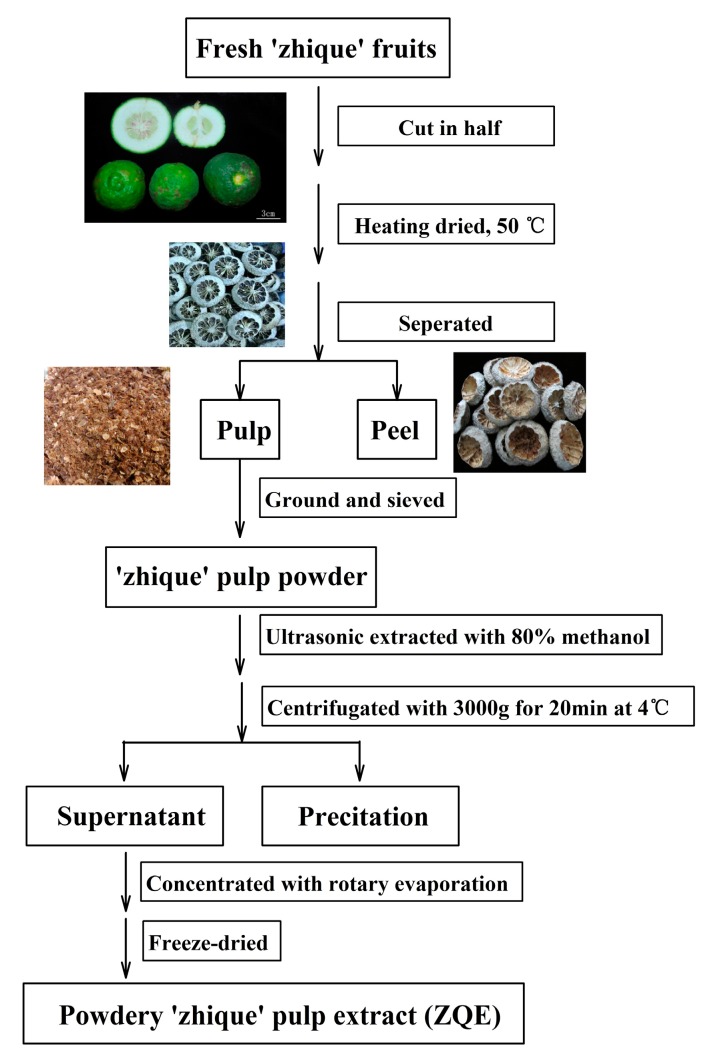
The extraction methods used to isolate ‘Zhique’ pulp extract (ZQE) from ‘Zhique’ fruits.

The procedure for ZQE preparation is outlined in Figure 1. ZQE was a very complex mixture containing 399 compounds detected by LC-ESI-MS/MS analysis, and the result is presented in detail in Figure S1 and Table S1. The qualitative compositions were classified into 22 categories of metabolites (Table 1), and flavonoids were the most abundant metabolites in the ZQE. Hence, we further analyzed the flavonoid profiles in ZQE by high performance liquid chromatography (HPLC).

### 2.2. Determination of Flavonoids Composition of the ZQE by HPLC

**Figure 2:**
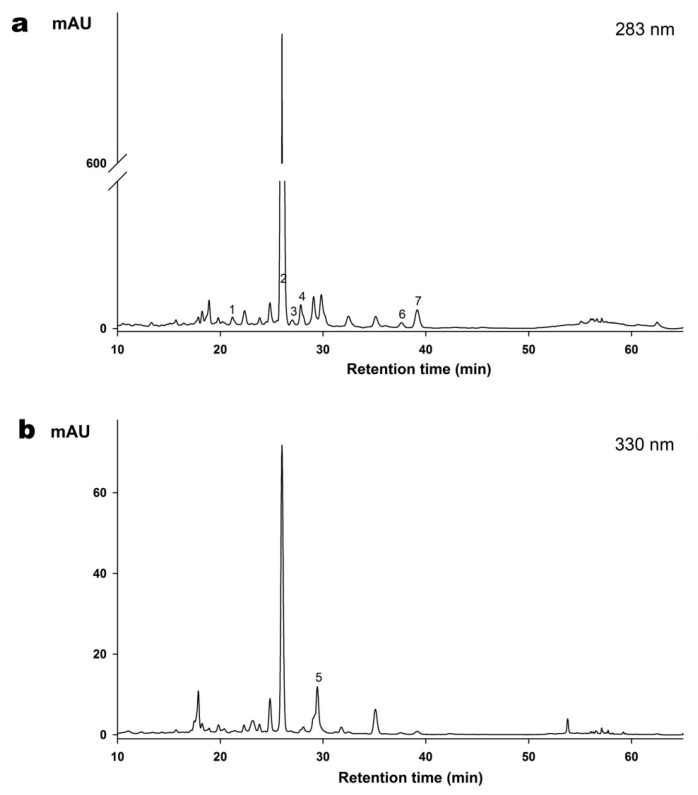
High performance liquid chromatography (HPLC) analysis with detection at 283 nm (**a**) and 330 nm (**b**) of the ZQE. Samples were analyzed using 15–75% methanol in 0.3% formic acid for 70 min. 1: Eriocitrin, 2: Naringin, 3: Hespiridin, 4: Neohesperidin, 5: Rhoifolin, 6: Naringenin, 7: Poncirin.

The chromatographic fingerprint of gradient reversed phase HPLC analyses with different detection wavelengths (283 nm and 330 nm) for ZQE are exhibited in Figure 2. Seven flavonoids were quantified in this study (Table 2). It is worth noting that naringin was the most abundant flavonoid in ZQE. More specifically, the absorption peak area of naringin accounted for 70% when the detection wavelength was 283 nm and the concentration was 79.59 ± 0.21 mg/g in ZQE. Meanwhile, the content of other flavonoids was lower, and naringenin (1117.82 ± 21.78 μg/g) was the least abundant among the identified compositions.

### 2.3. Effects of ZQE on Cell Viability in RAW 264.7 Macrophages

**Figure 3:**
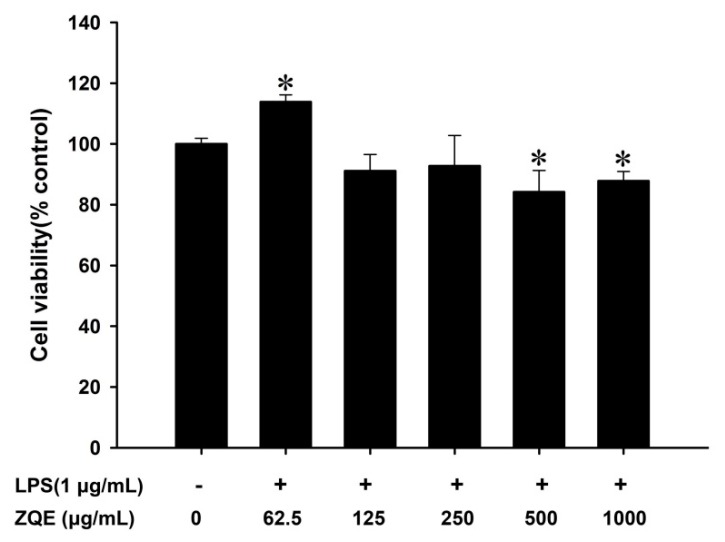
Effects of ZQE on cell viability in RAW 264.7 macrophages. The cells were treated with ZQE (0, 62.5, 125, 250, 500, and 1000 μg/mL) in the presence of LPS (1 μg/mL) for 18 h. Cell viability was measured by a MTT assay. The data shown are from three independent experiments expressed as mean ± SD. * *p* < 0.05 compared to the control (without ZQE and LPS). LPS: lipopolysaccharide; MTT: Methylthiazolyldiphenyl-tetrazolium bromide.

The cell viability of the ZQE was evaluated in RAW 264.7 macrophages cells by a methylthiazolyldiphenyl-tetrazolium bromide (MTT) assay. The results showed that low levels of ZQE had no inhibitory effect on cell viability (≤250 μg/mL), while high levels of ZQE markedly reduced cell viability by 15.78% and 12.17% at concentrations of 500 and 1000 μg/mL, respectively (Figure 3).

### 2.4. Effects of ZQE on the Production of PGE_2_ in LPS-Induced RAW 264.7 Macrophages

**Figure 4:**
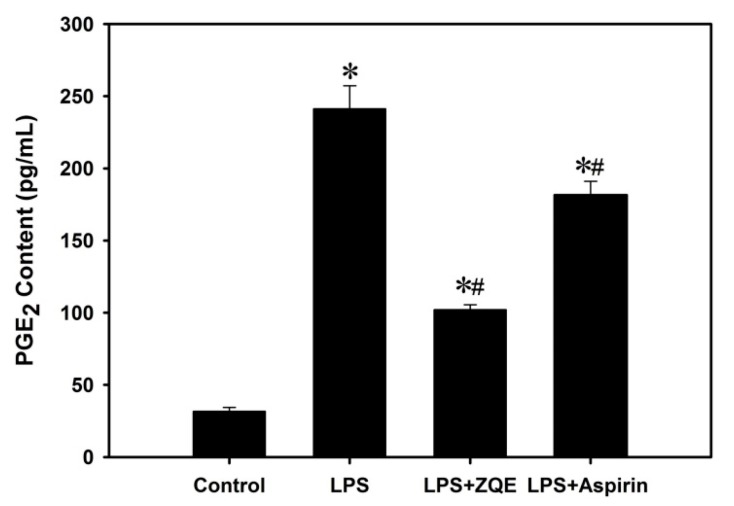
Effects of ZQE on the PGE_2_ production in LPS-induced RAW 264.7 macrophages. The cells were pretreated with ZQE (250 μg/mL) and aspirin (250 μg/mL) in the presence of LPS (1 μg/mL) for 12 h, and aspirin was used as a positive control. The production of PGE_2_ in the supernatant were assayed using ELISA kits. The data shown are from three independent experiments and expressed as mean ± SD. * *p* < 0.05 and # *p* < 0.05 compared the control (with no LPS, ZQE, or aspirin) and LPS alone, respectively. LPS: lipopolysaccharide; PGE_2_: Prostaglandins E2; ELISA: enzyme-linked immunosorbent assay.

RAW 264.7 macrophages cells were co-treated with ZQE (250 μg/mL) or aspirin (250 μg/mL) and LPS (1 μg/mL) for 12 h, and both the supernatants and cells were then collected. As shown in Figure 4, LPS significantly triggered the production of PGE_2_ (241.32 ± 15.90 pg/mL) compared with the control group (31.73 ± 2.72 pg/mL), while ZQE at 250 μg/mL significantly inhibited the production of PGE_2_ in LPS-induced RAW 264.7 macrophages. Moreover, the values of PGE_2_ in the ZQE-treated groups were lower than aspirin, a positive control group.

### 2.5. Effects of ZQE on Expression of Pro-Inflammatory Cytokines in RAW 264.7 Macrophages

**Figure 5:**
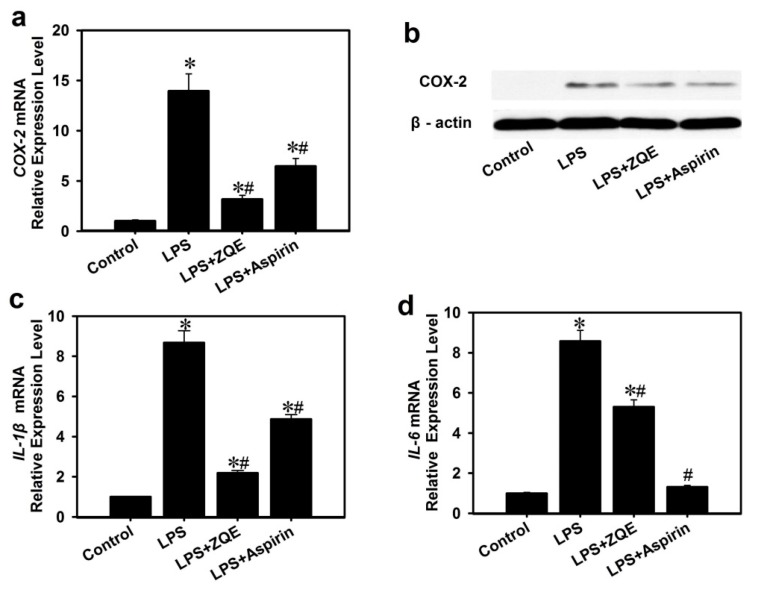
Effects of ZQE on LPS-induced COX-2, IL-1β and IL-6 expression in RAW 264.7 macrophages. The concentration of LPS is 1 μg/mL, and aspirin was used as a positive control, and the concentrations of ZQE and aspirin are both 250 μg/mL. (**a**) COX-2 mRNA expression; (**b**) COX-2 protein expression, β-actin as a loading control; (**c**) IL-1β mRNA expression; (**d**) IL-6 mRNA expression. The data shown are from three independent experiments and expressed as mean ± SD. * *p* < 0.05 and # *p* < 0.05 compared the control (with no LPS, ZQE, or aspirin) and LPS alone, respectively. COX-2, cyclooxygenase-2; LPS, lipopolysaccharide; PGE_2_, Prostaglandins E2; IL-1β, interleukin 1β; IL-6, interleukin 6.

The results in Figure 5a demonstrated that the COX-2 mRNA expression level in LPS-treated macrophage cells was increased by ~14-fold compared to the control group. However, application of ZQE significantly diminished the effects of LPS induction of COX-2 mRNA expression. Simultaneously, the values in ZQE-treated groups were remarkably less than the values in aspirin-treated groups.

Furthermore, COX-2 expression at the protein level was analyzed by Western blot as shown in Figure 5b. Similarly, application of ZQE (250 μg/mL) and aspirin (250 μg/mL) partially suppressed the COX-2 protein expression induced by LPS in RAW 264.7 macrophages. The results indicated that ZQE attenuated LPS-induced COX-2 secretion in RAW 264.7 macrophages.

To further examine the anti-inflammatory activity of ZQE, pro-inflammatory cytokines, IL-1β and IL-6 were determined at mRNA levels in LPS-induced RAW 264.7 macrophages. Compared with the control group, the expression of IL-1β (~nine-fold) and IL-6 (~nine-fold) mRNA of LPS (1 μg/mL) group was significantly increased (Figure 5c,d). Supplementation of ZQE (250 μg/mL) or aspirin (250 μg/mL) with LPS significantly reduced mRNA expression of IL-1β and IL-6 compared with the LPS-only group.

### 2.6. Effects of ZQE on the Gene Expression of Inflammatory Mediators and Cytokines in LPS-Induced Primary BMDCs

**Figure 6:**
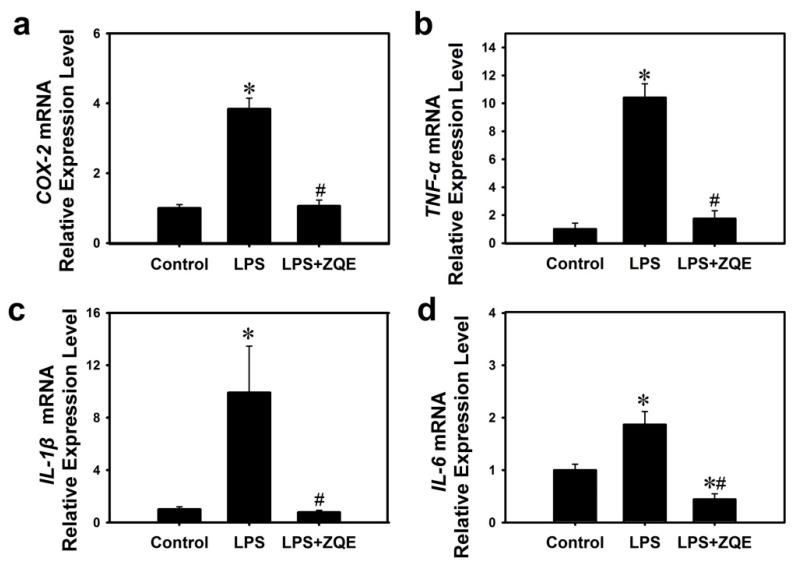
Effects of ZQE on LPS-induced mRNA expression of COX-2, TNF-α, IL-1β, and IL-6 (**a**–**d**) in primary BMDCs. The concentrations of LPS and ZQE is 100 ng/mL and 1 μg/mL, respectively. The data shown are from three independent experiments and expressed as mean ± SD. * *p* < 0.05 and # *p* < 0.05 compared the control (with no LPS or ZQE) and LPS alone, respectively. LPS: lipopolysaccharide; COX-2: cyclooxygenase-2; TNF-α: tumor necrosis factor-alpha; IL-1β: interleukin 1β; IL-6: interleukin 6; BMDCs: bone marrow-derived dendritic cells.

To further confirm the anti-inflammatory activity of ZQE, primary mouse BMDC cells were treated by LPS, with or without ZQE. The results in Figure 6 revealed that LPS (100 ng/mL ) markedly increased the transcriptional expression of COX-2 (3.84-fold), IL-1β (9.91-fold), IL-6 (1.87-fold)and TNF-α (10.42-fold), compared with the control group (cell culture medium only). However, ZQE at as low as 1 μg/mL completely abolished the effect of LPS on induction of inflammation in primary mouse dendritic cells. In the group of LPS + ZQE, the IL-6 level was even lower than the basal level of the control group.

## 3. Discussion

Natural products have the characteristics of low toxicity and outstanding pharmacological effects. Many literatures have reported that natural products or Chinese herbal medicines have good anti-inflammatory activity. The methanol extract of *Ardisiatinctoria* can prevent a LPS-induced inflammatory response through a decrease in the production levels of nitric oxide and PGE_2_, and the down-regulation of IL-1β and IL-6 in RAW 264.7 macrophages, and had no effect on cell viability at a concentration of 0–40 µg/mL [21]. *Muntingia calabura* leaves extracts have anti-inflammatory activity against ethanol-induced gastric ulceration in rats [22]. It was reported that aqueous and 70% ethanolic extracts of Exocarpium *Citri grandis* (*C. grandis*, Huajuhong in Chinese) have the clear effect of reducing the cough linked to inflammation in NIH mice [23]. In the present study, ZQE was one kind of natural plant product extracts, exhibiting low cytotoxicity in LPS-stimulated RAW 264.7 macrophages. ZQE at the concentration of 62.5 μg/mL did not inhibit the RAW 264.7 macrophages cell viability, suggesting little side-effects. Importantly, the results revealed that ZQE also exhibited strong anti-inflammatory activity in the LPS-induced RAW 264.7 macrophage stable cell line and mouse primary BMDCs through inhibition and down-regulation of the secretion and expression of inflammatory cytokines and mediators, such as PGE_2_, IL-6, and TNF-α, especially in primary BMDCs.

Previous studies indicate that the mixture or single flavonoid or polyphenol compounds extracted from citrus plants have anti-inflammatory activities in vivo and in vitro [11,13,19]. Flavonoids are a group of the main components of polyphenols and are widely present in citrus. In the present study, a large amount of flavonoids were detected in ZQE, and naringin was predominant; this result was consistent with previous research on *Citrus wilsonii* Tanaka [9]. Flavonoids are pharmacologically effective in attenuating inflammation [10,11,24]. Numerous studies have suggested that citrus flavonoids have a positive effect in blocking the inflammatory response in vivo [12,13] and in vitro [23,25,26]. According to previous studies and the present results, naringin may play a major role in the anti-inflammatory process in macrophages and dendritic cells. Growing evidence has indicated that naringin exerts anti-inflammatory roles in vivo [27,28,29] and in vitro [19], and naringin is considered as a protective agent against diseases related to inflammation, such as Parkinson’s disease [30,31]. Naringin extracted from *Citrus grandis* ‘Tomentosa’ can reduce the production of chemokines induced by LPS in the RAW 264.7 macrophage cell line [19]. In addition, it was also found that naringin can mitigate LPS-induced acute lung injury in mice through blocking the nuclear factor kappa B (NF-κB) signaling pathway [28], and it has obvious anti-inflammatory effects on chronic pulmonary neutrophilic inflammation in cigarette smoke-exposed rats by attenuating the secretion of inflammatory cytokines, such as TNF-α and interleukin-8 [29]. Moreover, our studies demonstrated that naringin is rich in ZQE. So, naringin is believed to play a major role in anti-inflammatory activity in the present study, and this needs to be further confirmed in the next experiment.

Aspirin, also named acetylsalicylic acid, is a typical non-steroidal anti-inflammatory medicine, and was used as a positive control in the study. Aspirin was initially used for the treatment of rheumatism. Recently, it has also been used for cardiovascular disease [32,33,34]. Aspirin exerts its role in anti-inflammation via COX-2 and the NF-κB signaling pathway [35,36]. In this study, aspirin exhibited the inhibitory effects on LPS-induced inflammation in RAW 264.7 macrophages. ZQE had a comparable effect to aspirin. However, non-steroidal anti-inflammatory drugs, such as aspirin which are used for the treatment of inflammatory diseases, have side effects on gastric mucosa, kidneys, bronchi, and the cardiovascular system [37]. Comparing an equal concentration of ZQE and aspirin, the ZQE exhibited greater effects on the suppression COX-2, IL-1β, and IL-6 expression than aspirin. Therefore, it could be possible to consider ZQE as a potential therapeutic agent for future study.

LPS acts as a prototypical endotoxin by binding the CD14/toll-like receptor 4/myeloid differential protein 2 receptor complex in dendritic cells, macrophages, monocytes, and B cells, etc., which promotes inflammation by the secretion of pro-inflammatory cytokines and other biological compounds [38]. LPS-promoted production of inflammatory mediators, including IL-1β, IL-6, and TNF-α, is related to the NF-κB signaling pathway. Meanwhile, aberrant expression of COX-2, a pro-inflammatory enzyme, leads to elevated levels of PGE_2_. In the present study, LPS obviously irritated the secretion of IL-1β, IL-6, and TNF-α, and markedly provoked the production of PGE_2_ and expression of COX-2 at mRNA and protein levels. ZQE not only targeted the production of PGE_2_, but also participated in the transcriptional and post-transcriptional regulation of COX-2 against inflammation induced by LPS. These results were consistent with the previous study [13]. We found that ZQE suppressed the transcriptional expression of IL-1β, IL-6 and TNF-α in LPS-stimulated macrophages and dendritic cells. Therefore, the results demonstrated that ZQE had significant anti-inflammatory activity in LPS-triggered inflammation and the mechanism may be related to NF-κB signaling pathway which warrants further studies.

In the present study, there are two cell models, RAW 264.7 macrophage stable cell line and primary mouse BMDCs, used to investigate the anti-inflammatory property of the extract from ‘Zhique’ pulp. The former cell line stimulated by LPS is common used as an in vitro model in studies related to inflammation or inflammatory diseases [13,39,40,41]. While dendritic cells play an important role in the initiation of adaptive immune responses, and are main used in the studies ofimmunity [42,43] and also involved in inflammation study [44,45]. The inflammation response were quite obvious in primary BMDCs after co-treated with LPS (100 ng/mL) and ZQE (1 μg/mL). The mRNA expression of COX-2, TNF-α, IL-1β, and IL-6 were inhibited significantly compared with LPS group. This phenomenon powerfully proves that ZQE has strong anti-inflammatory characteristics.

Recently, the phytochemical profiles and the antioxidant activity of *Citrus wilsonii* Takana were paid much attention [8,9]. In traditional horticulture, *Citrus wilsonii* Takana, a wild citrus species, has commonly been used as a rootstock for mandarin, due to its resistance to freezing temperatures and diseases, which improves the yield and quality of citrus fruit from the scion [46]. The present study demonstrated that extracts of ‘Zhique’ (*Citrus wilsonii* Takana) fruits had a very low cytotoxicity but excellent anti-inflammatory activity in LPS-induced RAW 264.7 macrophages and/or primary BMDCs. Thus, the ‘Zhique’ fruits do have unique potential medicinal value for the treatment of chronic inflammatory diseases.

## 4. Materials and Methods

### 4.1. Reagents

Hesperidin, neohesperidin, eriocitrin, narigenin, and poncirin were purchased from Yifang S&T Co. (Tianjin, China). Rhoifolin and naringin were purchased from Jinbao Online S&T Co. (Beijing, China). Methanol and formic acid were bought from Thermo Fisher Scientific Inc. (Waltham, MA, USA). LPS was obtained from Sigma-Aldrich Co. (St. Louis, MO, USA). MTT and dimethyl sulfoxide (DMSO) were purchased from Google Biotechnology Co. (Wuhan, China). Aspirin was obtained from Huanghai Pharmaceutical Co., Ltd., (Qingdao, China). Enzyme-linked immunosorbent assay (ELISA) kits for prostaglandins E2 (PGE_2_) were purchased from Bogu Biotechnology Co. (Shanghai, China).

### 4.2. Extraction of ‘Zhique’ Pulp Extract

A large batch of the ‘Zhique’ fruits at the fruit development stage (approximately 90 days after anthesis) was collected from farms in Nanzheng county (located in the Southern Qinling Mountains) in Shaanxi Province, China, and were identified by Prof. Shu’ang Peng (Huazhong Agriculture University, Wuhan, China). After harvest, the fruits were washed with distilled water, cut in half, dried at 50 °C, and then divided into peel and pulp. The pulp were ground and sieved through a 40-mesh sieve. The obtained powders were stored at −80 °C for the study.

Ten grams of ‘Zhique’ pulp powder was macerated in 1000 mL 80% methanol with double distilled water, and extracted for 30 min by ultrasonication. The combined extract solutions were centrifuged at 3000× *g* for 20 min at 4 °C and the supernatants were harvested, then concentrated with a rotary evaporator and freeze-dried. The resulting powders (approximately 3 g), named as ‘Zhique’ extract (ZQE), were stored at −20 °C for further experiments.

### 4.3. Analysis of the ZQE Profile by LC-ESI-MS/MS Analysis

ZQE was dissolved with 80% methanol to 10 mg/mL, filtered with a 0.22 μM nylon membrane, and then temporarily stored at 4 °C before analysis.

The separation of ZQE by ultra performance liquid chromatography (UPLC) was performed on a Shim-pack UFLC SHIMADZU CBM30A system with an ACQUITY UPLC HSS T3 C18 (2.1 mm × 100 mm, 1.8 µm) (Waters, MA, USA) column. Separation was accomplished at 40 °C with water (0.04% acetic acid) (A) and acetonitrile (0.04% acetic acid) (B) at 0.40 mL/min using a gradient program: 0–11.0 min, 100–5% A; 11.0–12.0 min, 5% A; 12.0–12.1 min, 5–95% A; 12.1–15.0 min, 95–95% A. The injection volume was 5 μL. The effluent was alternatively connected to an electrospray ionization (ESI)-triple quadrupole-linear ion trap (Q TRAP)-MS.

Linear ion trap (LIT) and triple quadrupole (QQQ) scans were acquired on a Q-TRAP mass spectrometer, the API 4500 Q TRAP LC/MS/MS System, equipped with an ESI Turbo Ion-Spray interface, operated in a positive ion mode, and controlled by Analyst 1.6 software (AB Sciex). The ESI source operation parameters were as follows: ion source: turbo spray; source temperature: 550 °C; ion spray voltage (IS): 5500 V; ion source gas I (GSI), gas II (GSII), and curtain gas (CUR) set at 55, 60, and 25.0 psi, respectively; the collision gas (CAD) was high. Instrument tuning and mass calibration were performed with 10 and 100 μmol/L polypropylene glycol solutions in QQQ and LIT modes, respectively. QQQ scans were acquired as multiple reaction monitoring (MRM) experiments with collision gas (nitrogen) set to 5 psi. Declustering potential (DP) and collision energy (CE) for individual MRM transitions was done with further DP and CE optimization. A specific set of MRM transitions were monitored for each period according to the metabolites eluted within this period.

### 4.4. Analysis of Flavonoids in ZQE

ZQE was pretreated as above (See Section 4.3) before HPLC analysis. The flavonoids were determined by reverse-phase analytical HPLC with an Agilent 1200 series system (Agilent Technology, Santa Clara, CA, USA). A two-solvent gradient mobile phase system was used, consisting of water with 0.3% formic acid (A) and absolute methanol (B). The gradient program was as follows: 0–10 min, 85–80% A; 10–15 min, 80–70% A; 15–25 min, 70–60% A; 25–48 min, 60–55% A; 48–55 min, 55–25% A; 55–60 min, 25% A; 60–65 min, 25–85% A; and 65–70 min, 85% A. The flow rate was set at 1 mL/min; the column temperature was 35 °C; and 10 μL of sample was injected into the Agilent HPLC system. Eriocitrin, naringin, hesperidin, neohesperidin, naringenin, and poncirin were detected at 280 nm, and rhoifolin was measured at 330 nm. The flavonoids were identified by comparing their retention time and the spectral characteristics of peaks with those of standards, and quantified based on the peak area of the sample and the corresponding standard.

### 4.5. Cell Culture

The RAW 264.7 macrophages were cultured as previous described [47]. The cells were grown in Dulbecco’s Modified Eagle’s Medium (DMEM) with high glucose (4.5 g/L) (Hyclone, GE Healthcare, Little Chalfont, UK) containing 10% fetal bovine serum (FBS) supplemented with 1% penicillin and streptomycin at 37 °C and 5% CO_2_ −95% air under humidified conditions.

Primary mouse BMDCs were obtained and cultured as described [48]. BMDCs were isolated from the femurs of the C57BL/6J mice. The cells were cultured in DMEM containing 20% FBS, supplemented with 1% penicillin and streptomycin and 10 μM β-mercaptoethanol with 20 ng/mL granulocyte-macrophage colony stimulating factor for BMDC differentiation. The medium was replaced every two days. During Days 7–9, BMDCs were harvested and stained with CD11c (BD Biosciences, 557401), and subjected to flow cytometry to confirm purity. BMDCs with purity greater than 90% was used for subsequent analysis.

### 4.6. Measurement of Cell Viability

The growth inhibitory effect of ZQE at concentrations ≤1000 μg/mL on RAW 264.7 cells was measured by a MTT assay, according to the manufacturer’s instructions. In brief, the RAW 264.7 macrophages were routinely cultured in a 96-well microplate at an initial density of 1 × 10^4^ cells/well for 24 h. Then the cells were co-treated with ZQE at different concentrations (0, 62.5, 125, 250, 500, or 1000 μg/mL) and LPS (1 μg/mL) for additional 18 h under normal cell culture conditions. Of note, ZQE and LPS were dissolved in DMEM. Then, 20 µL of MTT solution was added to each well and further incubated for additional 4 h at 37 °C. Finally, the cell culture medium was discarded and 100 μL DMSO was added to each well. The optical density was determined at 570 nm using a microplate reader (Tecan M200 pro, Grodig, Switzerland). All experiments were carried out in four biological replicates.

### 4.7. Measurement of PGE_2_

To access the effect of ZQE on the production of pro-inflammatory mediator PGE_2_ in the LPS-induced RAW 264.7 macrophages, the cells were stimulated with ZQE (250 μg/mL) or aspirin (250 μg/mL, as a positive control) and LPS (1 μg/mL) for 12 h, and the cell culture supernatants were collected. The content of PGE_2_ in the supernatants was measured using an ELISA kit according to the manufacturer’s protocol. The absorbance at 450 nm was recorded using a microplate reader.

### 4.8. RNA Isolation and qPCR Analysis

The RAW 264.7 macrophages were co-treated with ZQE (250 μg/mL) or aspirin (250 μg/mL) and LPS (1 μg/mL) for 12 h, while the primary BMDCs with ZQE (1 μg/mL) and LPS (100 ng/mL) for 8 h, respectively, to measure the mRNA expression of COX-2, IL-1β, IL-6, and TNF-α. Total RNA was isolated from these cell samples according to the manufacturer’s instruction of TRIpure reagent (Aidlab, Beijing, China), and then the first-strand copy DNA (cDNA) was synthesized using a reverse transcription kit (Takara, Japan). Quantitative real-time polymerase chain reaction (qPCR) was performed in an ABI 7500 Real-Time System with SYBR Green PCR Master Mix (Takara). Reactions were initiated with an initial incubation at 50 °C for two minutes and 94 °C for 10 min, followed by 40 cycles of 94 °C for 5 s, 60 °C for 15 s, and 72 °C for 10 s. The relative gene expression levels were calculated using the 2^−ΔΔCt^ method. The gene primers for qPCR are provided in Table 3. β-actin was used as an internal reference gene between different samples.

### 4.9. Western Blot Analysis

The RAW 264.7 macrophages were pre-incubated for 24 h, and subsequently treated with LPS (1 μg/mL) in the presence or absence of ZQE (250 μg/mL) or aspirin (250 μg/mL) for 12 h. Cells were washed three times with ice-cold phosphate-buffered saline (pH 7.4) and harvested with a cell scraper (Sigma-Aldrich, St. Louis, MO, USA). Whole-cell extracts were isolated in Radio immunoprecipitation assay lysis buffer with 0.1 % protease inhibitor cocktails on ice for 30 min, and the supernatant was harvested for Western blotting after 12,000× *g* centrifugation at 4 °C for 5 min. The protein quantitation was measured by a bicinchoninic acid assay with bovine serum albumin as a standard. Proteins were separated in a 10% sodium salt-polyacrylamide gel, and then transferred onto polyvinylidene difluoride membranes (Millipore, MA, USA). The blots were blocked with a 5% skim milk solution in Tris-buffered saline buffer with 0.01% Tween 20 (TBST) (Solarbio, Beijing, China) for 1 h, then incubated with anti-COX-2 antibody (1:1000 dilutions) in TBST at 4 °C for overnight, and then washed three times with TBST buffer on an orbital shaker. Finally, the membranes were incubated with horseradish peroxidase-linked anti-goat immune globulin G (1:3000 dilutions) secondary antibodies for 30 min and washed three times with TBST. Detection was performed using enhanced chemiluminescence.

### 4.10. Statistical Analysis

Data obtained from the experiments were expressed as mean ± standard deviation (SD). The statistical significance of the difference between groups was evaluated by Duncan in SPSS 19.0 (SPSS Inc., Chicago, IL, USA). *p* < 0.05 was considered to be a significant difference.

## 5. Conclusions

In summary, ZQE exhibited potent anti-inflammatory activities, such as suppressing PGE_2_ production in LPS-treated RAW 264.7 macrophages, and attenuating the TNF-α transcription in LPS-treated primary BMDCs. ZQE played inhibitory roles in LPS-induced inflammation in RAW 264.7 macrophages and primary BMDCs, mainly through down-regulation of pro-inflammatory cytokines and chemokines. This study may pave a new way for developing of ZQE/‘Zhique’ as a potential alternative drug for treating chronic inflammatory diseases.

## Figures and Tables

**Table 1:** Profile of ZQE.

Compounds Classification	Numbers
Amino acids	28
Amino acid derivatives	22
Alkaloids	10
Anthocyanin	2
Carbohydrates	6
Catechin derivatives	3
Cholines	7
Coumarins and their derivatives	2
Flavonoids	65
Indoles and their derivatives	4
Lipids-fatty acid	8
Lipids-glycerides	2
Lipids-glycerophospholipid	17
Nucleotides and their derivatives	29
Organic acids and their derivatives	11
Terpenoids	4
Tryptamines and their derivatives	12
Phenolamides	13
Phenylpropanoids	14
Proanthocyanidins	2
Phytohormones	6
Vitamin and Vitamin-related	16

**Table 2:** Seven flavonoids compounds concentrations in the extract of ZQE ^a^.

Compounds	ZQE
Naringin (mg/g)	79.59 ± 0.21
Eriocitrin (μg/g)	2197.54 ± 18.29
Hespiridin (μg/g)	3348.21 ± 204.00
Neohesperidin (μg/g)	1909.71 ± 2.81
Rhoifolin (μg/g)	2818.0± 18.00
Naringenin (μg/g)	1117.82 ± 21.78
Poncirin (μg/g)	2114.42 ± 91.72

^a^ Data are reported as mean ± standard deviation (*n* = 3).

**Table 3:** Primer sequences used in quantitative real-time polymerase chain reaction (qPCR) analysis.

Gene Name	Forward/Reverse Primer Sequence (5′–3′)
β-actin	TGAAGGGCATCTTGGGCTACAC TGGGTGGTCCAGGGTTTCTTAC
COX-2	ATCTGGCTTCGGGAGCACAAC GAGGCAATGCGGTTCTGATACTG
IL-1β	GTTGACGGACCCCAAAAGAT CCTCATCCTGGAAGGTCCAC
IL-6	ACAAAGCCAGAGTCCTTCAGA TCCTTAGCCACTCCTTCTGT
TNF-α	ACTGAACTTCGGGGTGATCG TCTTTGAGATCCATGCCGTTG

## Supplementary Materials

Supplementary materials are available online.
