# Supplementary figures and images for: The Anti-Inflammatory Properties of Citrus wilsonii Tanaka Extract in LPS-Induced RAW 264.7 and Primary Mouse Bone Marrow-Derived Dendritic Cells
Source: Molecules. 2017 Jul 19;22(7):1213. doi: 10.3390/molecules22071213 (PMC6152223; doi:10.3390/molecules22071213)

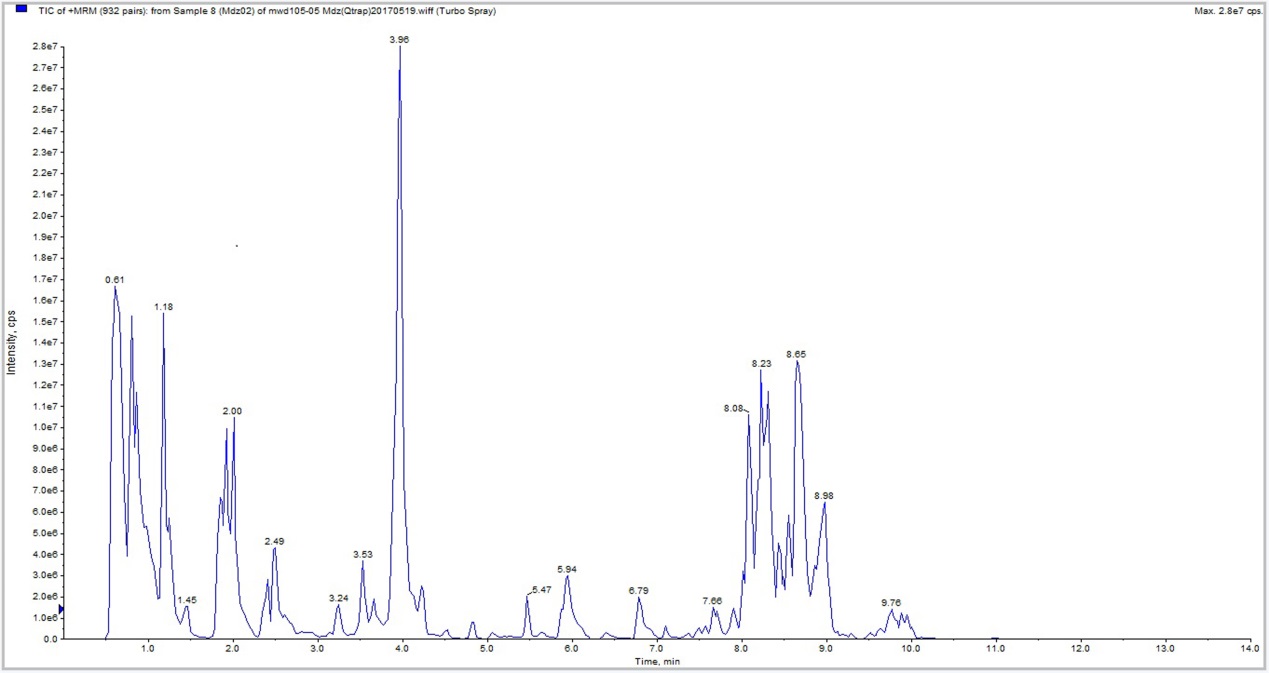


**Figure S1. Mass spectrometry total ion chromatogram of ‘Zhique’ pulp extract (ZQE) by LC-ESI-MS/MS.**

Supplement: Supplementary file 1 [file molecules-22-01213-s001.zip › Figure S1.docx]
